# The Effects of Hydro-Priming and Colonization with *Piriformospora indica* and *Azotobacter chroococcum* on Physio-Biochemical Traits, Flavonolignans and Fatty Acids Composition of Milk Thistle (*Silybum marianum*) under Saline Conditions

**DOI:** 10.3390/plants11101281

**Published:** 2022-05-10

**Authors:** Iraj Yaghoubian, Mohammed Antar, Saeid Ghassemi, Seyed Ali Mohammad Modarres-Sanavy, Donald L. Smith

**Affiliations:** 1 Department of Agronomy, Tarbiat Modares University, Tehran P.O Box 14115-336, Iran; iraj.yaghoubian@mail.mcgill.ca (I.Y.); modaresa@modares.ac.ir (S.A.M.M.-S.); 2 Department of Plant Science, McGill University, Montreal, QC H9X3V9, Canada; mohammed.antar@mail.mcgill.ca; 3 Department of Ecophysiology, University of Tabriz, Tabriz 5166616471, Iran; saeid.ghassemi67@gmail.com

**Keywords:** adenosine triphosphate, *Azotobacter chroococcum*, flavonolignans, milk thistle, oil, *Piriformospora indica*, salt stress, seed priming

## Abstract

Salinity is an important challenge around the world, effecting all physiological and biochemical processes of plants. It seems that seed priming can diminish the negative impacts of salinity. To study the effects of hydro-priming and inoculation with *Piriformospora indica* (Pi) and *Azotobacter chroococcum* (Az) on physio-biochemical traits, flavonolignans and fatty acids composition of milk thistle under saline conditions, a greenhouse experiment was carried out. Our results indicated that under salinity, seed priming, especially Pi, improved physio-biochemical properties in milk thistle. Under 120 mM NaCl, inoculation with Pi increased membrane stability index (MSI) and relative water content (RWC) (by 21.86 and 33.43%, respectively). However, peroxidase (POX) (5.57- and 5.68-fold in roots and leaves, respectively), superoxide dismutase (SOD) (4.74- and 4.44-fold in roots and leaves, respectively), catalase (CAT) (6.90- and 8.50-fold in roots and leaves, respectively) and ascorbate peroxidase (APX) (5.61- and 5.68-fold in roots and leaves, respectively) activities increased with increasing salinity. Contrary to salinity, hydro-priming with Az and Pi positively altered all these traits. The highest content of the osmolytes, adenosine triphosphate (ATP) content and rubisco activity were recorded in Pi treatments under 120 mM NaCl. Stearic acid (20.24%), oleic acid (21.06%) and palmitic acid (10.48%) increased, but oil content (3.81%), linolenic and linoleic acid content (22.21 and 15.07%, respectively) decreased under saline conditions. Inoculations of Pi positively altered all these traits. The present study indicated that seed priming with Pi under 120 mM NaCl resulted in maximum silychristin, taxidolin, silydianin, isosilybin, silybin and silymarin of milk thistle seeds.

## 1. Introduction

For more than 2000 years, our ancestors remedied gastrointestinal disorders and hepatitis using milk thistle (*Silybum marianum*) as a natural therapy; in particular, it is well-known for its liver-protective properties [1]. Through modulating specific proteins, milk thistle silymarin flavonolignan has anti-inflammatory, antioxidant and anti-metastatic activity [2]. Milk thistle seeds have a high vitamin E content and contain silybin, silymarin, silydianin, silychristin and phospholipids, the molecular structure of the flavonoids giving them unique properties [3,4]. This means they are effective against liver disease and help to retain healthy liver function [5]. Clinical confirmations are available for *S. marianum’s* hepatoprotective and anticarcinogenic activities. Breast and skin cancer, kidney cirrhosis, cervical cell and prostate problems are also treated with milk thistle [6]. The plant is commercially grown in Iran, China, Egypt, Canada, Pakistan, Poland, Uzbekistan and other European countries due to increasing demand for silymarin by the pharmaceutical industry. In recent decades, environmental stresses have been serious deterrents to the achievement of agricultural products, such as medicinal and crop products, all around the world [7]. Milk thistle cultivation is influenced by both biotic and abiotic stresses. One of the destructive abiotic effects which greatly reduces plant efficiency and growth is salinity [8]. While salty water was previously thought to be unsuitable for watering, study efforts over the recent decades have resulted in the implementation of some major irrigation schemes that rely on saline water [9]. The biochemical processes related to photosynthesis are affected directly by drought stress, generally through reductions in the influx of carbon dioxide into the stomata when closed or partially closed in order to limit leaf water loss and associated tissue dehydration. Therefore, the production of photosynthetic materials can be disrupted by drought stress, causing these materials to accumulate in leaves, which may restrict photosynthesis [10,11]. Enzymatic and non-enzymatic mechanisms are two defensive approaches of plants to counteract oxidative stress associated with photosynthesis disruption. Ascorbate, tocopherol, carotenoids and flavonoids are part of the non-enzyme system [12]. Previous findings show that widespread use of chemical fertilizers can have adverse effects on beneficial soil enzymes in the long term, therefore the use of non-chemical treatments such as hydro-priming and growth-promoting microorganisms can reduce the negative effects of chemical fertilizers [13].

Seed priming has been an effective technique for improving physiological traits and secondary metabolite biosynthesis in medicinal plants, which have been confirmed in pennyroyal [14] and wild mint [15]. Hydro-priming is a promising technique that allows for direct field sowing while also improving seed physiological efficiency and addressing the issue of weak stand establishment [16]. Bourioug et al. [17] reported that hydro-priming in sunflower improved all growth parameters and yield components under stress conditions. Field researches indicate that hydro-priming has the ability to increase emergence and stand establishment by shortening the growth period, from the planting of seed to the emergence of seedlings [18]. Farooq et al. [19] have expressed that although hydro-priming leads to beginning the initial stages of the seed germination process via seed hydration, the emergence of radical does not happen.

*Azotobacter* is the first known heterotrophic bacteria that is soil-borne, nitrogen-fixing, and belongs to the *Azotobacteraceae* family. These obligate aerobe bacteria are chemoorganotrophs, meaning they can provide energy in the form of fat stores, as well as carbon from organic matter [20]. Seed priming with an *Azotobacter chroococcum* consortium is efficacious to elevate the plant growth and performance indicators in a variety of ways, both directly and indirectly [21].

Plants, mainly their roots, are colonized by fungi [22]. The process begins with the biotrophic invasion of nearby alive cells, followed by a cell-death period in which the fungus kills the cells of the root in order for them to develop themselves in the host plant before proliferating [23]. As a result of its extensive hyphal network, the endophyte promotes nutrient uptake quality [24]. *Aspergillus sydowii*, *Sebacina vermifera*, *Pseudomonas fluorescens*, *Gaeumannomyces graminis*, *Chlamydomonas reinhardtii*, *Rhizopus stolonifera* and *Aspergillus niger*, have all been proved to interact with *Piriformospora indica* in the soil [25,26]. Many essential agricultural and horticultural plants have beneficial associations with *Piriformospora indica*, and this fungus not only allows host plants to thrive under nutrient-deficient soils, but also grants resistance to biotic (foliar and root pathogens) and abiotic (heavy metals, high temperature, cold, salinity, water and drought) stress, induces early flowering, improves seed production, regulates plant development, and increases the production of essential oils in medicinal plants [27,28].

There is a limited number of studies on the impact of hydro and nanoparticle priming on milk thistle plants growing under abiotic stresses [29,30]. However, not many studies are available on seed priming with PGPM on milk thistle under salinity stress. Considering the significant function of seed priming in lessening the detrimental impacts of abiotic stresses, we hypothesize that seed priming with hydro, *Azotobacter chroococcum* and *Piriformospora indica* can play a vital role in salinity tolerance in milk thistle.

In this study, we aimed to evaluate how seed priming with hydro, *Piriformospora indica* and *Azotobacter chroococcum* affected physio-biochemical parameters and secondary metabolites including flavonolignans and fatty acid profile in milk thistle grown under saline conditions.

## 2. Results

### 2.1. Relative Water Content (RWC)

**Figure 1:**
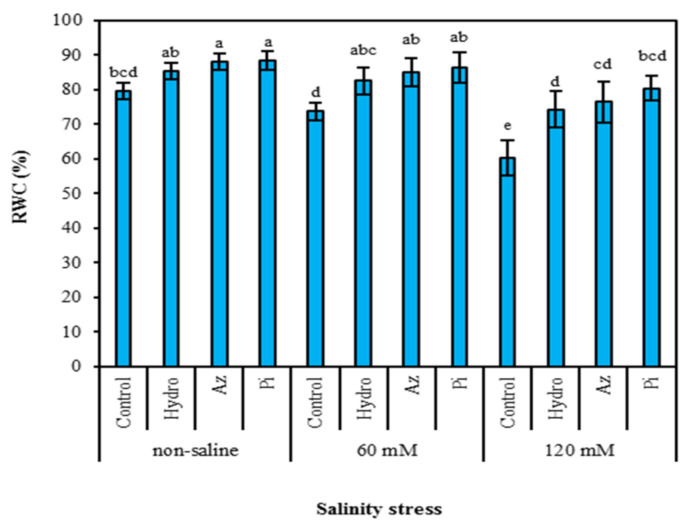
Effect of seed priming on relative water content (RWC) of milk thistle leaves under different levels of salinity. The same letter within each column indicates no significant difference among treatments (*p* ≤ 0.05) using New Duncan’s test. Values mean of four replicates ± SD. Hydro: hydro-priming, Az: *Azotobacter chroococcum* and Pi: *Piriformospora indica*.

Increasing salinity levels significantly reduced the amount of water relative content (RWC) in milk thistle. In contrast, seed priming, especially with Pi, increased leaf RWC in all levels of salinity. The two highest amounts of RWC (88.42% and 88.09%) were obtained in plants primed with Pi and Az, respectively, compared to control plants, for which RWC was 79.63% (Figure 1).

### 2.2. Membrane Stability Index (MSI)

**Figure 2:**
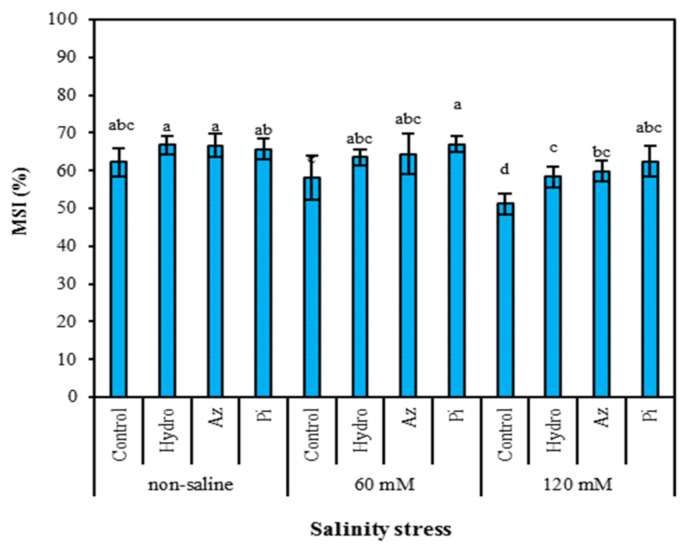
Effect of seed priming on membrane stability index (MSI) of milk thistle leaves under different levels of salinity. The same letter within each column indicates no significant difference among treatments (*p* ≤ 0.05) using New Duncan’s test. Values mean of four replicates ± SD. Hydro: hydro-priming, Az: *Azotobacter chroococcum* and Pi: *Piriformospora indica*.

Membrane damage increased with increasing level, so membrane stability index (MSI) was negatively impacted by stress. However, seed priming with Pi mitigated the negative effect of salinity on the MSI in leaves (Figure 2). Our results showed that the highest MSI was obtained in Pi priming (66.94%) under 60 mM salinity, hydro-priming (66.73%) and Az (66.69%) in non-saline treatments.

### 2.3. Adenosine Triphosphate (ATP) Content

**Figure 3:**
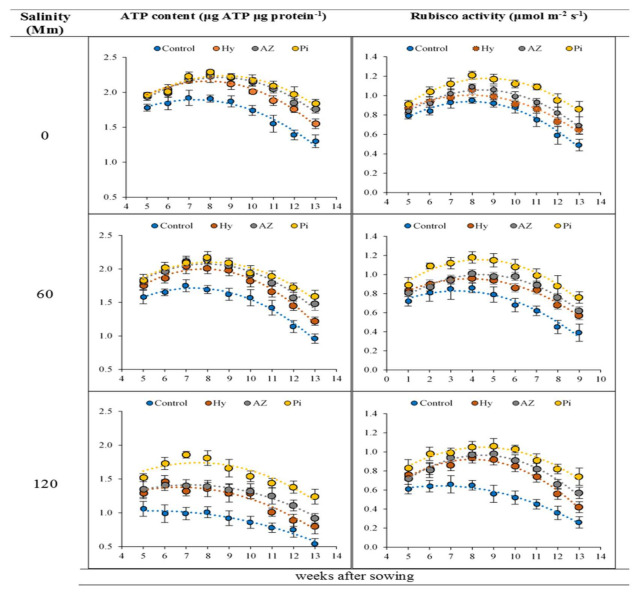
Changes in ATP content and rubisco activity in milk thistle leaves in response to salinity stress (0, 60 and 120 mM) and seed priming. Values mean of four replicates ± SD. Hydro: hydro-priming, Az: *Azotobacter chroococcum* and Pi: *Piriformospora indica*.

Milk thistle leaves showed an increase in adenosine triphosphate (ATP) content when treated with hydro-priming, Az and Pi, and this decreased after 7–8 weeks. Salt stress decreased the ATP content in all plants. Seed treatment increased ATP content in the leaves. Pi had greater positive effect on leaf ATP content than other treatments (Az, hydro-priming and control treatments) (Figure 3).

### 2.4. Rubisco Activity

Rubisco activity was reduced by increasing salt stress in all treatments, while the activity was increased with time of soaking. Rubisco activity declined after 8 weeks of soaking, and it was lowest in week 13 of the experiments in treated and control treatments. Seeds treated with hydro-priming, Az and Pi under control and salt conditions had increased rubisco activity. In particular, Pi showed the greatest increase in rubisco activity (Figure 3).

### 2.5. Malondialdehyde (MDA) Concentration

**Figure 4:**
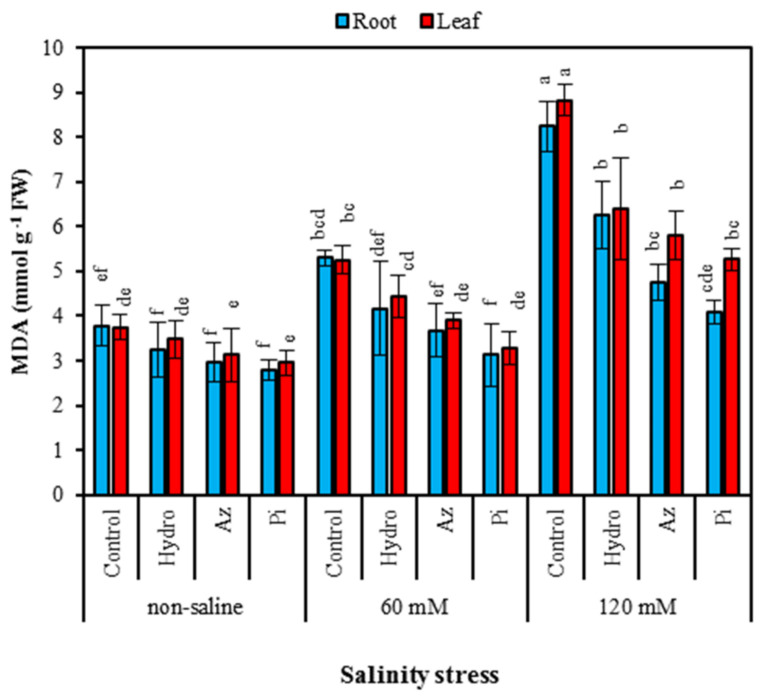
Effect of seed priming on malonyl dialdehyde (MDA) content of milk thistle root and leaves under different levels of salinity. The same letter within each column indicates no significant difference among treatments (*p* ≤ 0.05) using New Duncan’s test. Values mean of four replicates ± SD. Hydro: hydro-priming, Az: *Azotobacter chroococcum* and Pi: *Piriformospora indica*.

In all plants, the content of malondialdehyde (MDA) under salinity stress increased in roots and leaves by 2.18- and 2.35-fold, respectively. However, seed priming decreased this variable at all salinity levels. The maximum MDA content in roots was 8.23 mmol g FW^−1^, whereas leaves recorded 8.82 mmol g FW^−1^ in 120 mM control plants (Figure 4).

### 2.6. Antioxidant Enzyme Activities

**Figure 5:**
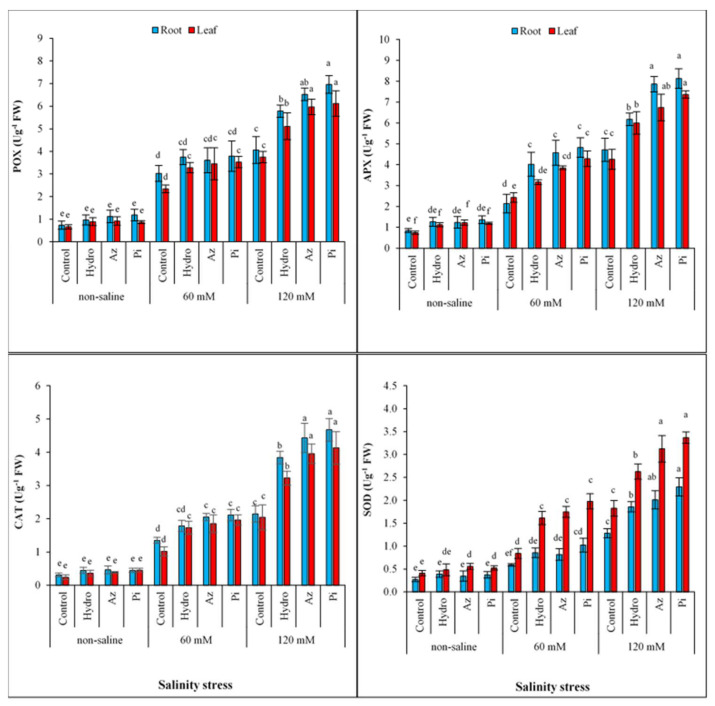
Effect of seed priming on peroxidase (POX), ascorbate peroxidase (APX), catalase (CAT) and superoxide dismutase (SOD) activities in root and leaves of milk thistle under different salinity levels. The same letter within each column indicates no significant difference among treatments (*p* ≤ 0.05) using New Duncan’s test. Values mean of four replicates ± SD. Hydro: hydro-priming, Az: *Azotobacter chroococcum* and Pi: *Piriformospora indica*.

Catalase (CAT), peroxidase (POX), superoxide dismutase (SOD) and ascorbate peroxidase (APX) activities in roots and leaves increased with increasing salinity condition in all treated and non-treated plants. Priming seeds with hydro, Az and Pi increased the activities of these enzymes under all salinity levels. Under all salinity and seed priming conditions, the CAT, POX and APX activities were greater in roots than leaves, but SOD activity was lesser in roots than leaves (Figure 5).

### 2.7. Soluble Sugar and Proteins

**Figure 6:**
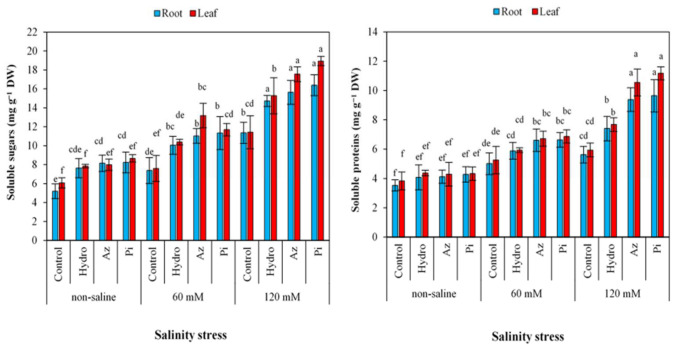
Effect of seed priming on soluble sugar and proteins in root and leaves of milk thistle under different salinity levels. The same letter within each column indicates no significant difference among treatments (*p* ≤ 0.05) using New Duncan’s test. Values mean of four replicates ± SD. Hydro: hydro-priming, Az: *Azotobacter chroococcum* and Pi: *Piriformospora indica*.

For all treated and non-treated plants, soluble sugar and protein contents in roots and leaves were increased by increasing salinity concentrations (Figure 6). Under the 120 mM NaCl condition, seed priming by Pi increased soluble sugar (by 30.71 and 39.40% in roots and leaves, respectively) and soluble protein (by 40.59 and 46.82%, in roots and leaves, respectively).

### 2.8. Proline and Glycine Betaine Contents

**Figure 7:**
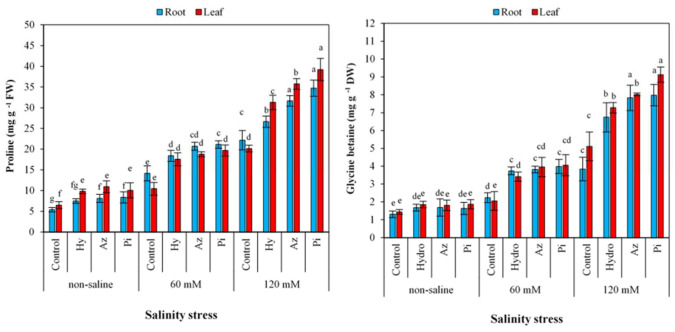
Effect of seed priming on proline and glycine betaine in root and leaves of milk thistle under different levels of salinity. The same letter within each column indicates no significant difference among treatments (*p* ≤ 0.05) using New Duncan’s test. Values mean of four replicates ± SD. Hydro: hydro-priming, Az: *Azotobacter chroococcum* and Pi: *Piriformospora indica*.

The contents of proline and glycine betaine (Figure 7) in root and leaves increased by seed priming and salinity. Seed priming with Pi increased proline under non-saline conditions (35.56 and 35.68% in roots and leaves, respectively), 60 mM NaCl (32.93 and 46.94% in roots and leaves, respectively) and 120 mM NaCl (36.44 and 48.67% in roots and leaves, respectively). Under 120 mM NaCl, seed priming with hydro, Az and Pi increased glycine betaine in roots (42.94, 50.89 and 51.82%, respectively) and leaves (29.71, 36.20 and 44.03%, respectively).

### 2.9. Flavonolignans in Milk Thistle Seed

Among the flavonolignans, the content of silymarin was higher than other constituents. Flavonolignans of milk thistle seeds such as silychristin (32.29%), taxidolin (44.12%), silydianin (8.63%), isosilybin (52.91%), silybin (36.88%) and silymarin (37.04%) were significantly increased as a result of salt stress. These flavonolignans were enhanced by seed priming, mainly with Pi (Table 1).

### 2.10. Oil Content and Fatty Acids Composition

The oil content of milk thistle, for all plants primed with hydro, Az and Pi, increased with increasing salinity levels, but for the control treatments, this variable decreased under severe NaCl conditions (120 mM). The major fatty acid observed in milk thistle seeds was linoleic acid at 50.68%, followed by oleic acid at 29.62%. We also detected linolenic acid, palmitic acid and stearic acid in *Silybum marianum* seeds (Table 2). In all plants, increasing the salinity levels increased palmitic acid, stearic acid and oleic acid levels, but linoleic acid and linolenic acid content decreased. Seed priming under saline and non-saline conditions decreased the palmitic acid, stearic acid and oleic acid contents; however, the linoleic acid and linolenic acid contents increased (Table 2).

## 3. Discussion

The relative water content (RWC) of plants was used to determine their resistance to salt stress [31]. A reduction in RWC in plants under NaCl stress (Figure 1) could be related to cation imbalance, sodium toxicities and osmotic stress, and it may be relevant to plant vigor reduction [32]. The potential of plants’ stomata to mitigate water loss and the potential of plants to absorb water through developed roots systems reflect plants’ resistance to salt conditions [33]. Seed priming with hydro-priming, *Azotobacter chroococcum* (Az) and *Piriformospora indica* (Pi) increased the RWC of milk thistle plants through improved biomass of shoot and root and increased root hair production [23]. Priming with Pi increased the RWC more than that of other treatments. Due to the endosymbiosis established in plant roots, the fungi have been allowed to change some mechanisms and metabolisms in the roots, resulting in increased growth indicators [34]. It is stated that communication between fungi and plants provide mutual benefits to both, which can increase the host plant’s resistance to salinity. This is because the uptake of minerals, the ratio of plant phytohormones and the amount of secondary compounds in plants will be affected by fungi [35].

The stability of cell membranes can be disrupted by high salt concentration [24], leading to increased membrane stability index (MSI) via leakage of ions (Figure 2). The enhancement of antioxidant enzyme activities by these treatments (Figure 5) was closely linked to the increase in MSI via seed priming (Figure 2). The positive impact of hydro-priming, Az and Pi on MSI of milk thistle plants (Figure 2) could be attributed to the maintenance of positive leaf turgor (Figure 1) and efficient and longer use of soil resources by plants under stress due to the early establishment of seedlings [36].

A reduction in ATP content (Figure 3) under stress conditions is related to decreasing magnesium (Mg^2+^) and calcium (Ca^2+^) contents in leaves, as these elements play a key role in ATP synthesis in plant cells by activating two basic enzymes (Ca-ATPase and Mg-ATPase) [37]. In addition, in saline environments, inhibiting PSII (Fv/Fm) electron transfer decreased the ATP content of leaves [37]. Zheng et al. [38] stated that the diminution in the synthesis of ATP in wheat is associated with PSII disruptions. Seeds treated with Pi and Az and hydro-priming had increased membrane stability indices (Figure 2), possibly through improving Mg^2+^ and Ca^2+^ contents in leaves and ATP content in leaf tissues, improved by increasing Fv/Fm [37].

The adverse impacts of salinity, such as reduced N_2_ content (structural element in enzyme body), K^+^ content, which is essential for enzyme activation, and ATP content (Figure 3), and increased ROS and oxidative stress under salt stress (Figure 5), significantly diminish rubisco enzyme production in milk thistle leaves (Figure 3). Pi and Az treatment and hydro-priming can shield photosynthetic proteins such as rubisco from degradation through improving the activities of antioxidants and decreasing the ROS. As a result, given that these seed treatments are able to restore salinity-induced disorder in photosynthetic processes, they might play a drastic role in the overall activity of photosynthesis [39].

Oxidative stress induced by salinity may result in excess reactive oxygen species (ROS) production, resulting in lipid peroxidation [40], which can be measured by using malondialdehyde (MDA) content [41]. Saline conditions can disrupt cell membranes (Figure 2) by leaking free radicals and depleting enzyme activities, resulting in lipid peroxidation and excess MDA production [42] in milk thistle roots and leaves. However, seed priming led to a slight reduction in MDA content in both treated and untreated seeds (Figure 4). Seed priming has the potential to reduce lipid peroxidation and increase salt tolerance [43]. Kamithi et al. [44] reported that several processes, including membrane repair and build-up, ATP synthesis, synthesis and activation of nucleic acids and several antioxidant enzymes and the cytoplasmic membrane repair, begin during priming. Thus, the content of MDA began to decrease during priming (Figure 4). Previously, research on potatoes indicated that the amount of MDA was reduced in hydro-primed plants [45]. MDA accumulation was low in plants inoculated with Az and Pi treatments, indicating the alleviation of salt stress through microbial treatments. MDA is predominantly produced via the degradation of polyunsaturated lipids effected via reactive oxygen species (ROS) [46,47]. Therefore, a strong link is formed between the observed resistance of Az and Pi-colonized milk thistle plants to salt stress and the ability of Az and Pi to inhibit the degradation of polyunsaturated lipids by inhibiting the formation of excess ROS [48].

The accumulation of ROS family members including superoxide anion (O_2•_^−^), hydroxyl radical (•OH) and H_2_O_2_ in plants are a physiological indication of the presence of salinity stress [49]. Under no circumstance should we be oblivious to antioxidant enzymes’ role in plants, which makes plants better able to overcome environmental stresses due to the critical function of metabolites in scavenging ROS [50]. Under stress conditions, antioxidant enzymatic activities, ascorbate peroxidase (APX), superoxide dismutase (SOD), catalase (CAT), and peroxidase (POX) increased with increasing salt levels (Figure 5), helping in the disappearance of ROS and hence reducing the oxidative stress activity [51]. Enzymatic activities lead to high levels of H_2_O_2_, which acts as a precursor for other ROS [52]. Increasing the antioxidant enzymes content (POX, CAT, APX and SOD) with hydro-priming, Az and Pi treatments leads to removal of ROS and a reduction in damage to cell membranes [53].

NaCl induced an increase in soluble sugar (Figure 6) and protein (Figure 6) contents both in roots and leaves of milk thistle plants. Carbohydrate variations are serious and in no way can we be oblivious to its changes, as physiological processes such as respiration, transportation, and photosynthesis rely on carbohydrates [54]. Under salinity stress conditions, several experiments have shown that plant sugar content increased [55]. Soluble sugars and proteins in plants treated with hydro-priming, Az and Pi (Figure 6) were greater than those of control plants, while the MDA content in non-treated plants was more than in treated ones (Figure 4). Hydro-priming’s role in improving soluble protein is also related to the dissolving of the b-subunit of the 11-S globulin storage protein [56]. Similarly, hydro-priming effectively mobilized compounds such as soluble sugars, proteins, and free amino acids from storage tissues into developing embryonic organs in pigeon pea (*Cajanus cajan*) [57].

In this study, increasing salinity levels resulted in higher amounts of proline (Figure 7) and glycine betaine contents (Figure 7). Osmolyte compound accumulation is commonly used to alleviate the adverse impacts of water limitations and drought and salt tolerance adaptive mechanisms [58]. Our findings indicate that hydro-priming and root colonization with Az and Pi increased the proline and glycine betaine accumulations. Accumulations of proline and glycine betaine are sensitive physiological indications of plant response to salt and other stresses. In response to salt stress, plant aggregates inorganic ions and organic solutes, glycine betaine, soluble sugars and proline to adjust osmotic pressure [59].

Environmental stresses have been shown to have a considerable influence on the agglomeration of secondary metabolites in plants [58]. Under unfavorable conditions, the amount of flavonoids and phenolic acids in milk thistle increased (Table 1). Afshar et al. [60] found the same dependence. Flavonoids contain antioxidant attributes due to their capability to remove free radicals, and they also have the ability to modulate cell signaling pathways. In this analysis, silymarin was the most abundant flavonoligan in milk thistle seeds, accounting for 18.91% of the total phenolic compounds (Table 1). Treatments with hydro-priming, Az and Pi have incremental effects on flavonolignans of milk thistle seeds (Table 1). Flavonoids are important secondary metabolites in plant defensive systems against biotic and abiotic stresses [61]. Moreover, a sizeable amount of polyphenols in plants grown under stress conditions can prevent excessive ROS accumulation and photoinhibition-induced harm [62].

Some salt-stressed plants exhibited increases in oil content (Table 2), which may be attributed to a decrease in primitive metabolites because of salt stress, allowing intermediate products to become eligible for secondary metabolite synthesis. In particular, the effects of salt stress on essential oils and their compounds might be attributed to their influence on metabolism and enzyme activity [63]. Hydro-priming and the inoculation of microbes also increased oil content. In a large number of plants, increasing salinity levels decreased contents of oleic acid, palmitic acid and stearic acid, but linolenic acid and linoleic acid contents decreased (Table 2). In a related investigation in *F. vulgare* and *Cymbopogon martini*, oil content was enhanced by Az treatment [64]. Additionally, Pi has been shown to enhance oil content in *Origanum majorana* and *Sesamum indicum* [65,66]. While the underlying cause for the increase or decrease in oil content and composition is unknown, it may be due to a protective reaction [58,65], morphological traits [67,68], up-regulation of biosynthetic genes [68] and P availability. Enhancement of commercial essential secondary metabolites has also been accomplished successfully using cell-culture methods aided by a fungal elicitor [58,69]. Although the underlying reasons for the increase in major oil components are unclear, it can be argued that better metabolism, control of phytohormones, and adjustment of biosynthetic genes may all contribute to the increase in a specific class of secondary metabolites. An elicitor of rhizobacteria and fungi can activate the pathway of biosynthetic [70], resulting in increased secondary metabolites in the cell; this was further confirmed through the activity of ammonia-lyase (PAL) activity, which acts as a limiter in syntheses of lignin secondary metabolite [71].

Our findings indicated that seed priming has positive effects on promoting milk thistle physio-biochemical characteristics, flavonolignans and fatty acids composition. Seed priming influences plant growth in a wide range of plants via a variety of direct and indirect mechanisms [72,73]. It can be argued that seed priming is a quite effective method for improving rapid emergence and achieving a high level of vigor, thus increasing growth under saline conditions.

## 4. Materials and Methods

### 4.1. Experimental Conditions

In 2019, a factorial greenhouse experiment with four replications, organized following a randomized complete block design, was carried out to investigate the impact of hydro-priming and innoculation with *Azotobacter chroococcum* (Az) and *Piriformospora indica* (Pi) on milk thistle (*Silybum marianum* L. var Budakalasz) under salinity conditions. In this experiment, 3 NaCl levels of salinity (0-control, 60 and 120 mM) and 4 levels of seed priming (control, hydro-priming, *Azotobacter chroococcum* and *Piriformospora indica*) were used.

Seeds of milk thistle were obtained from Pakan Bazr Company, Isfahan, Iran. Before sowing, the seeds were divided into four equal parts, and one of these parts was then considered as a control. Another part was soaked with water for 20 h at 20 °C and then dried to the initial moisture. The other two samples were inoculated with Az and Pi. Then, the 15 milk thistle seeds were sown at a depth of 4 cm in each plastic pot (23 cm diameter × 24 cm height) containing 10 kg soil. After germination, thinning was performed to keep only 4 plants in each pot. The soil used in this experiment was sandy loam with pH 7.1, EC 0.24 ds m^−1^, organic carbon (0.44%), nitrogen (0.07%), available phosphorus (31 mg kg^−1^) and available potassium (169 mg kg^−1^).

The greenhouse temperature ranged from 25 ± 2 °C during the day to 20 ± 2 °C at night, with a relative humidity of 50–60%.

*Azotobacter* was grown in 500 mL containers with 200 mL liquid Luria–Bertani (LB) medium. This medium was put in an incubator at 28 °C in 120 rpm for two days. In a few words, the bacterial suspensions prepared (equal to 108 CFU mL^−1^) was inoculated onto milk thistle seeds following Yaghoubian et al. [74].

For the cultivation of Pi, the Yaghoubian et al. [75] method was used. The fungus was cultured in a 25 mL modified Kafer solid medium in a Petri dish and placed in an incubator at 28 °C for 10 days. To encourage production of spores, the culture surface was scratched with a skimmer, then, about 15 mL distilled water containing 0.05% Tween-20 was added to the medium. Finally, the spores were centrifuged at 6000 rpm for 5 min at 4 °C, and when the density reached 8 × 10^8^ spores mL^−1^, seeds were inoculated by the spore suspensions for 6 h [58].

The pots were irrigated on 2-day intervals to the field-capacity moisture content (leaching was avoided). After 4 weeks of planting, salt stress was carried out by adding the appropriate concentration of NaCl solutions to the pots until the salt concentrations reached appropriate levels (60 and 120 mM). Control plants were well-watered throughout the experiment period. At the stage of flowering, samples were harvested for physiological and biochemical determinations. After physiological maturity, all seeds were harvested to measure the quality and nutritional properties of the seeds.

### 4.2. Relative Water Content (RWC) and Membrane Stability Index (MSI)

The water status of the examined plants in the maximum flowering stage was determined using RWC. Following the green weight (Gw) determination, the leaves were soaked in sterile water for 6 h to reach approximate saturation weight (Sw), and the leaves were dried at 60 °C for 24 h to determine the dry weight (Dw) [76]. RWC was calculated through the following Equation (1):

(1) $$\mathrm{RWC}=\left[\frac{\mathrm{Gw}-\mathrm{Dw}}{\mathrm{Sw}-\mathrm{Dw}}\right]\times 100$$

The membrane stability index (MSI) was calculated using method of Ghassemi et al. [77].

### 4.3. Adenosine Triphosphate (ATP) Content and Rubisco Activity

To measure adenosine triphosphate (ATP) content, we followed the method of Larsson and Olssonl [78], and for the assay we used a Luminometer (model: novalum, Charm Sciences, Lawrence, MA, USA).

For determination of rubisco activity in the mature seeds, we followed Lobo et al. [79], in which a spectrophotometer was adjusted to 340 nm to measure NADH oxidation. Further, 25 mM KHCO_3_, 100 mM bicine, 0.25 mM NADH, 20 mM MgCl_2_, 80 nuked creatine-phosphokinase, 3.5 mM ATP, 80 nkat 3-phosphoglyceric phosphokinase, 80 nkat G-3-P dehydrogenase and 5 mM phosphocreatine were used to make the assay buffer.

### 4.4. Malondialdehyde (MDA) Concentration

The Rao and Sresty [80] method was updated to calculate MDA levels. On ice, 300 mg of frozen milk thistle leaves and roots were homogenized in 0.1% trichloroacetic acid (TCA). After centrifuging the homogenate at 11,000 rpm for 15 min at 4 °C, the amount of absorption of the supernatant was measured at 600 nm in mmol g^−1^ FW.

### 4.5. Determination of Antioxidant Enzymes Activities

Frozen leaf and root samples of milk thistle were frozen in liquid nitrogen, ground into a fine powder and removed with 10 mL of 50 mM phosphate buffer, pH 7. In brief, extracted fluid was centrifuged for 30 min at 4 °C at 12,000 rpm. The supernatant was then purified and used to measure the actions of various antioxidant enzymes in a spectrophotometer. The superoxide dismutase (SOD) was calculated at 560 nm, and this was defined as SOD activity (one unit).

The activity of peroxidase (POX), ascorbate peroxidase (APX) and catalase (CAT) were assessed at 240, 290 and 470 nm, respectively. Following the method of Yaghoubian et al. [74], all the mentioned antioxidant enzymes have been reported as Ug^−1^ FW [81].

### 4.6. Soluble Sugar and Proteins

The anthrone reagent was used to calculate the total amount of soluble sugar [55]. In summary, 0.5 g of samples are homogenized and filtered in 95% ethanol. The residue was removed twice with 70% ethanol, and the filtrates were combined and centrifuged at 6000 rpm for 15 min. Then, 100 μL supernatant was applied, along with 3 mL anthrone reagent (100 mL H_2_SO_4_ 72% + 150 mg anthron) and the mixture was heated for 10 min in a bath at 100 °C. Using glucose as a blank, the liquid’s absorbance was estimated at 625 nm. The pH of the protein samples was adjusted following Yaghoubian et al. [74].

The protein content of the supernatant was evaluated after a sufficient dilution of the supernatant at 535 nm. Both sugar and protein quantities were reported as mg g^−1^ DW [74].

### 4.7. Proline Content and Glycine Betaine Content

Proline was obtained using 0.3 g of root and leaf and 6 mL of extraction medium, as described by Yaghoubian et al. [74]. A colorimetric reaction with ninhydrin was used to measure proline using spectrophotometry at 515 nm as mg g^−1^ FW. L-proline was used to construct a regular curve for measuring proline concentration.

A total of 500 mg of ground samples of leaves and roots was combined with 5 mL water and toluene (0.05%) to obtain the glycine betaine content in milk thistle. At 25 °C, tubes were shaken for two days. Potassium tri-iodide solution (0.1 mL) and 1 mL of 2 N HCl were applied to 0.5 mL of prepared sample and shaken for 90 min in an ice-cold water bath. The organic layer’s optical density was measured at 365 nm as mg g^−1^ DW [82].

### 4.8. Determination of Flavonolignans

Flavonolignans were measured using the procedure recommended by Sitaramaraju et al. [83]. A total of 5 g of ground milk thistle fruit was placed in the Soxhlet apparatus and petroleum ether for 5 h. The sample was air-dried before being moved to a flask and drained with 20.0 mL of methanol using a reflux condenser for 30 min. A rotary evaporator was used to evaporate the mixed methanol extracts under vacuum fully. Briefly, analyses were carried out using an Agilent Technologies 1100 liquid chromatograph: column temperature 25 °C; column: Lichrospher RP18 (0.250 m × 4 mm, 5 µm; Merck, Burlington, MA, USA).

### 4.9. Oil Extraction and Fatty Acids Composition

This section followed the AOCS method using a Soxhlet device for oil extraction from 3 g mature seeds of each plot in petroleum ether for five hours [84]. Each sample’s oil content was expressed as percentage.

Fatty acid analysis was performed using the PerkinElmer Clarus 500 GC method (PerkinElmer, Shelton, CT, USA) with a Supelco SP-2560 column (100 m by 0.25 mm by 0.2 m film thickness). The initial oven temperature was 110 °C and it was gradually raised to 190 °C at 3 °C min^–1^ for 15 min. The temperature was then raised at the same rate to 235 °C and kept there for 10 min. A final temperature rise of 2 °C min^–1^ was added before the temperature reached 240 °C. The injector and detector (FID) were adjusted to 250 °C. The injection mechanism was set to a split ratio of 20:1, and a 0.5 µL sample was injected.

### 4.10. Statistical Analysis

Statistical Analysis Software (SAS Institute Inc., Cary, NC, USA, 2004, version 9.3) was used for data analysis of variances. Experimental data were analyzed using two-way ANOVA, and Duncan’s multiple range test at *p* ≤ 0.05 was used to compare means of each variable. The figures were drawn using Excel software.

## 5. Conclusions

This study aimed at understanding the potential positive effects of seed priming on adverse effects of salinity on bio-physiological traits, flavonolignans and finally fatty acid composition. The findings obtained in this study have provided new insights on the effects of hydro-priming and PGPM on the growth of milk thistle under salinity. Clearly, the study has indicated that salinity can have both positive and negative effects on bio-physiological traits. Furthermore, adenosine triphosphate (ATP) content and rubisco activity decreased in leaves of milk thistle with increasing salinity levels. However, antioxidant enzyme activities and osmolyte contents in root and leaves of milk thistle plants increased with increasing salinity. Additionally, flavonolignans and the oil content of milk thistle seeds were significantly increased as a result of salt stress. Hydro-priming and inoculation with Az or Pi, especially Pi, improved the physio-biochemical properties, greatly reduced the negative effects of salinity stress and improved the oil quality of milk thistle. It would also be of interest to study the interactions between the studied treatments that caused positive effects.

## Figures and Tables

**Table 1:** Effect of seed priming and salinity on flavonolignans of milk thistle seed (mg·g^−1^ DM).

Salinity	Seed Priming	Silymarin	Silybin	Isosilybin	Silydianin	Taxidolin	Silychristin
Non-saline	Control	16.28 ± 1.88 e	9.11 ± 0.22 e	2.21 ± 0.28 h	1.02 ± 0.08 f	0.68 ± 0.10 e	0.96 ± 0.12 d
	Hydro	18.18 ± 0.39 cde	10.13 ± 0.87 de	2.82 ± 0.22 fg	1.12 ± 0.10 f	0.74 ± 0.08 de	0.01 ± 0.10 cd
	Az	17.49 ± 1.66 de	10.08 ± 0.81 de	2.72 ± 0.35 gh	1.28 ± 0.16 def	0.77 ± 0.03 cde	1.08 ± 0.05 cd
	Pi	18.94 ± 0.98 cde	10.49 ± 0.69 cde	2.96 ± 0.21 efg	1.41 ± 0.19 def	0.83 ± 0.05 bcd	1.18 ± 0.17 bcd
60 mM	Control	18.14 ± 2.17 cde	10.65 ± 0.75 cde	2.66 ± 0.10 gh	1.24 ± 0.04	0.75 ± 0.04 de	1.04 ± 0.15 cd
	Hydro	20.63 ± 1.97 bcd	12.06 ± 0.91 bcd	3.84 ± 0.11 cd	1.45 ± 0.21 def	0.95 ± 0.09 bc	1.16 ± 0.11 bcd
	Az	20.96 ± 0.79 bcd	12.18 ± 1.85 bcd	3.51 ± 0.31 de	1.66 ± 0.35 cde	0.91 ± 0.12 bcd	1.24 ± 0.21 bcd
	Pi	21.38 ± 2.90 bc	12.82 ± 0.67 b	3.76 ± 0.33 cd	1.82 ± 0.27 c	1.02 ± 0.11 b	1.37 ± 0.13 b
120 mM	Control	22.31 ± 1.26 b	12.47 ± 0.87 bc	3.38 ± 0.54 def	1.72 ± 0.32 cd	0.98 ± 0.04 b	1.27 ± 0.12 bc
	Hydro	27.44 ± 1.92 a	16.01 ± 1.48 a	4.24 ± 0.14 bc	2.36 ± 0.31 b	1.37 ± 0.11 a	1.68 ± 0.25 a
	Az	28.05 ± 2.16 a	16.43 ± 2.07 a	4.56 ± 0.37 ab	2.71 ± 0.29 ab	1.41 ± 0.14 a	1.79 ± 0.06 a
	Pi	29.84 ± 2.64 a	17.52 ± 1.71 a	4.83 ± 0.45 a	2.84 ± 0.36 a	1.52 ± 0.22 a	1.94 ± 0.20 a

The same letter within each column indicates no significant difference among treatments (*p* ≤ 0.05) using New Duncan’s test. Values mean of four replicates ± SD. Hydro: hydro-priming, Az: *Azotobacter chroococcum* and Pi: *Piriformospora indica.*

**Table 2:** Effect of seed priming and salinity on oil content and fatty acids composition of milk thistle seed (%).

Salinity	Seed Priming	Oil Content	Palmitic Acid	Stearic Acid	Oleic Acid	Linoleic Acid	Linolenic Acid	UI
Non-saline	Control	30.14 ± 3.12 cd	7.82 ± 0.35 b	5.68 ± 0.14 c	29.62 ± 0.62 def	50.68 ± 0.57 bc	0.45 ± 0.03 ab	1.32 ± 0.03 ab
	Hydro	31.27 ± 2.02 cd	7.71 ± 0.27 b	5.52 ± 0.41 c	29.02 ± 0.52 ef	51.13 ± 0.88 ab	0.47 ± 0.05 ab	1.33 ± 0.03 ab
	Az	34.49 ± 2.62 bc	7.75 ± 0.25 b	5.41 ± 0.20 d	28.11 ± 1.08 f	51.95 ± 0.43 ab	0.49 ± 0.06 a	1.33 ± 0.02 ab
	Pi	34.83 ± 1.29 bc	7.81 ± 0.41 b	5.47 ± 0.34 cd	28.04 ± 1.09 f	52.34 ± 0.55 a	0.49 ± 0.07 a	1.34 ± 0.02 a
60 mM	Control	32.20 ± 3.29 c	8.02 ± 0.47 ab	5.97 ± 0.64 bc	30.71 ± 0.31 bc	48.46 ± 0.94 d	0.41 ± 0.03 abc	1.28 ± 0.01 b
	Hydro	37.45 ± 2.71 a	7.92 ± 0.27 b	5.73 ± 0.35 c	29.51 ± 0.85 def	50.51 ± 1.22 bc	0.44 ± 0.02 ab	1.32 ± 0.02 ab
	Az	38.84 ± 3.55 a	7.81 ± 0.15 b	5.52 ± 0.17 c	28.48 ± 0.69 ef	50.62 ± 0.44 bc	0.45 ± 0.04 ab	1.31 ± 0.02 ab
	Pi	39.18 ± 2.16 a	7.75 ± 0.53 b	5.54 ± 0.45 c	28.22 ± 0.94 f	50.45 ± 0.44 bc	0.47 ± 0.04 ab	1.30 ± 0.03 ab
120 mM	Control	26.28 ± 0.88 d	8.64 ± 0.32 a	6.83 ± 0.26 a	35.86 ± 1.22 a	43.04 ± 1.74 e	0.35 ± 0.02 c	1.23 ± 0.02 c
	Hydro	37.05 ± 1.54 ab	8.11 ± 0.42 ab	6.40 ± 0.35 ab	31.54 ± 1.28 b	48.72 ± 0.51 d	0.40 ± 0.03 bc	1.30 ± 0.02 ab
	Az	37.51 ± 1.89 a	8.02 ± 0.30 ab	5.98 ± 0.15 bc	30.13 ± 0.36 bcd	49.48 ± 0.37 cd	0.43 ± 0.04 ab	1.30 ± 0.03 ab
	Pi	38.11 ± 2.19 a	7.93 ± 0.29 b	5.86 ± 0.16 bc	29.98 ± 0.33 cde	50.02 ± 1.02 cd	0.48 ± 0.03 ab	1.31 ± 0.03 ab

The same letter within each column indicates no significant difference among treatments (*p* ≤ 0.05) using New Duncan’s test. Values mean of four replicates ± SD. Hydro: hydro-priming, Az: *Azotobacter chroococcum* and Pi: *Piriformospora indica.*

## Data Availability

The data presented in this study are available on request from the corresponding author. The data are not publicly available due to the privacy statement in the original project.
